# Tagging and Capturing of Lentiviral Vectors Using Short RNAs

**DOI:** 10.3390/ijms221910263

**Published:** 2021-09-23

**Authors:** Martin Panigaj, Michael P. Marino, Jakob Reiser

**Affiliations:** 1 Faculty of Science, Institute of Biology and Ecology, Pavol Jozef Safarik University in Kosice, 041 54 Kosice, Slovakia; martin.panigaj@gmail.com; 2 Center for Biologics Evaluation and Research, Division of Cellular and Gene Therapies, U.S. Food and Drug Administration, Silver Spring, MD 20993, USA; Yorick73@cox.net

**Keywords:** lentiviral vectors, vector engineering, pseudotyping, vector targeting, nucleic acid aptamers

## Abstract

Lentiviral (LV) vectors have emerged as powerful tools for transgene delivery ex vivo but in vivo gene therapy applications involving LV vectors have faced a number of challenges, including the low efficiency of transgene delivery, a lack of tissue specificity, immunogenicity to both the product encoded by the transgene and the vector, and the inactivation of the vector by the human complement cascade. To mitigate these issues, several engineering approaches, involving the covalent modification of vector particles or the incorporation of specific protein domains into the vector’s envelope, have been tested. Short synthetic oligonucleotides, including aptamers bound to the surface of LV vectors, may provide a novel means with which to retarget LV vectors to specific cells and to shield these vectors from neutralization by sera. The purpose of this study was to develop strategies to tether nucleic acid sequences, including short RNA sequences, to LV vector particles in a specific and tight fashion. To bind short RNA sequences to LV vector particles, a bacteriophage lambda N protein-derived RNA binding domain (λN), fused to the measles virus hemagglutinin protein, was used. The λN protein bound RNA sequences bearing a boxB RNA hairpin. To test this approach, we used an RNA aptamer specific to the human epidermal growth factor receptor (EGFR), which was bound to LV vector particles via an RNA scaffold containing a boxB RNA motif. The results obtained confirmed that the EGFR-specific RNA aptamer bound to cells expressing EGFR and that the boxB containing the RNA scaffold was bound specifically to the λN RNA binding domain attached to the vector. These results show that LV vectors can be equipped with nucleic acid sequences to develop improved LV vectors for in vivo applications.

## 1. Introduction

Over the past two and a half decades, lentiviral (LV) vectors have emerged as powerful tools for transgene delivery [1]. LV vectors have been tested in numerous clinical trials involving ex vivo transduced cells, such as hematopoietic stem cells and mature T cells [2,3]. Four LV vector-based gene therapy products have received regulatory approval, including Kymriah (tisagenlecleucel) for the treatment of relapsed/refractory B cell acute lymphoblastic leukemia (B-ALL) [4], Zynteglo (betibeglogene autotemcel) for the treatment of transfusion-dependent beta-thalassemia [5], Breyanzi (lisocabtagene maraleucel) for the treatment of relapsed or refractory large B cell lymphoma (LBCL) [6] and Abecma (idecabtagene vicleucel) for the treatment of adult patients with relapsed or refractory multiple myeloma [7].

LV vectors are also applied directly in vivo for therapeutic purposes. A non-primate LV vector system based on equine infectious anemia virus (EIAV) has been investigated clinically to treat ocular disorders [8]. In another in vivo application, an HIV-1-based, integration-deficient LV vector expressing the NY-ESO-1 cancer testis antigen targeted to dendritic cells was used to promote an immune response against NY-ESO-1-expressing tumors [9].

The design of LV vectors to allow the targeted transduction of specific cell types in vivo has been challenging and the efficacy of such vectors can be affected by neutralizing antibodies. Virus engineering approaches have been critical in helping to address some of these issues [10,11,12]. For example, a number of pseudotyping strategies aimed at broadening the tropism of LV vectors to transduce previously nonpermissive cells or to replace the vector’s tropism in order to transduce specific target cells exclusively have been described [13]. Pseudotyped retroviral vector particles bear envelope glycoproteins derived from other enveloped viruses and acquire the tropism of the virus from which the glycoprotein was derived [12,14,15,16]. A versatile strategy for LV vector targeting involving engineered measles virus (MV) hemagglutinin (H) and fusion (F) glycoproteins has emerged [12,17,18]. Various protein ligands, including epidermal growth factor (EGF) [19], IL-13 [20], single-chain antibodies [21] and designed ankyrin repeat proteins (DARPins) [22] have been successfully displayed using MV H, allowing retargeted LV vector delivery.

Engineering strategies aimed at the enhancement of the purification of LV vectors have also been investigated. For example, Yu et al. [23] used a library screening approach to identify variants of the vesicular stomatitis virus G protein (VSV-G) bearing hexahistidine tags, allowing the purification of LV vectors using Ni-nitrilotriacetic acid (NTA) affinity chromatography. Similarly, biotin has been displayed on the surface of LV vectors via the metabolic biotinylation of a low-affinity nerve growth factor receptor (LNGFR) domain incorporated on the vector’s surface, allowing the capture of vector particles by immobilized streptavidin and elution, via the addition of biotin [24].

Surface modification approaches have also been used to dampen innate immune responses to LV vectors [25]. LV vectors pseudotyped with VSV-G have long been known to be sensitive to complement-mediated inactivation by human serum [26]. Recent work by Munis et al. has revealed that alternative LV vector pseudotypes involving heterologous vesiculovirus G glycoproteins may ultimately allow the circumvention of this issue [27]. Furthermore, the co-display of complement-regulatory proteins on LV particles, especially decay accelerating factor (DAF)/CD55, has been shown to confer a significant level of protection against complement attack [28].

Other approaches to the modification of the surface of LV vectors have included direct covalent modification. Shielding of VSV-G-pseudotyped LV vectors by poly(ethylene) glycol (PEG) conjugation (PEGylation) was shown by Croyle et al. [29] to increase the resistance of these vectors to complement-mediated inactivation. An alternative approach to the shielding of VSV-G pseudotyped LV vectors, involving a thin polymer shell synthesized in situ onto the vector’s envelope, was described by Liang et al. [30]. The shielded vector possessed enhanced stability in the presence of human serum, indicating protection of the vector by the polymer shell from human serum complement inactivation.

The goal of this study was to develop novel approaches to tether short RNA sequences to LV vector particles in a specific fashion, using RNA binding proteins. The bacteriophage λN protein-boxB system [31] provided a promising solution to this problem. The λN protein, fused to the MV H protein displayed on the LV vector particles, enabled the capture of vector particles by using magnetic beads via an RNA scaffold containing a boxB sequence, as well as the transduction of target cells.

## 2. Results

### 2.1. Design of RNA Scaffolds Capable of Binding to a Bacteriophage Lambda N Protein

To attach RNA sequences to LV vector particles, a 22 amino acid RNA binding domain (λN) derived from the bacteriophage lambda antitermination protein N [31,32] was used. The λN protein has the capacity to bind to a 19 nucleotide RNA target sequence, referred to as the boxB RNA motif [31].

In our approach, the λN domain bound to a boxB sequence present on a scaffold RNA [33]. The scaffold RNA was in turn base-paired to a specific short RNA sequence, such as an RNA aptamer. A number of RNA scaffolds based on the design of the AriBo tag scaffold [33] were designed and tested. These RNA scaffolds are referred to as Ab2b, Ab2bA, Aab, Ab and bA, respectively. The scaffold RNAs were generated by in vitro transcription, using the DNA templates shown in Figure S1. They contained either one or three copies of the boxB RNA motif [31] to mediate the binding of the scaffolds to a λN domain [34]. The scaffolds used also contained a complementary sequence to allow the binding of the RNA sequences via base pairing.

### 2.2. Testing the Ability of RNA Scaffolds to Bind to the H-λN2 Domain Displayed on Transfected Cells

**Figure 1:**
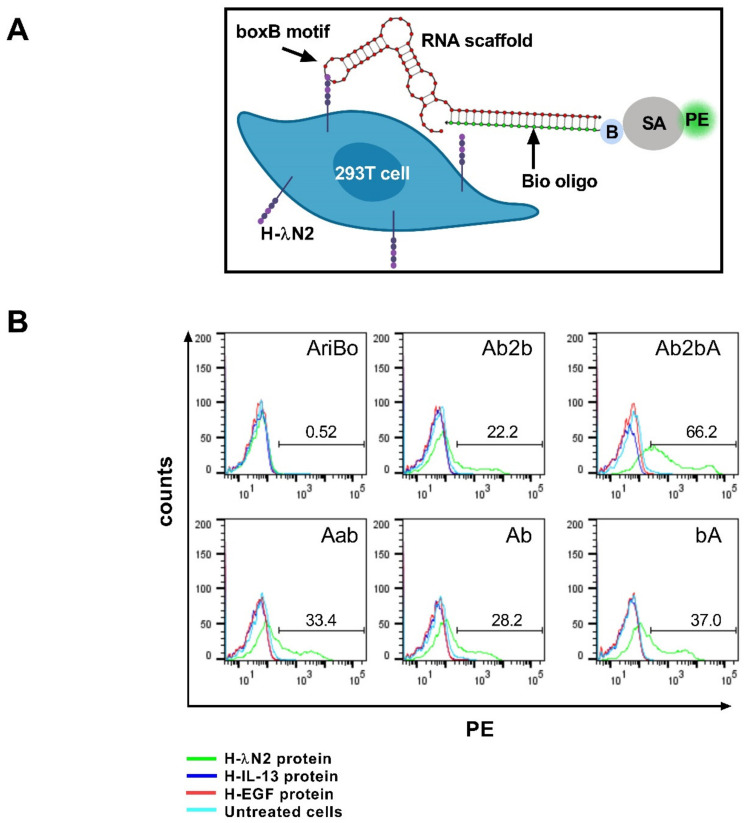
RNA scaffold binding to the H-λN2 protein, a RNA binding domain derived from the bacteriophage lambda antitermination protein N fused to the MV H protein in transfected HEK 293T cells. (**A**) Outline of the approach to test the binding of RNA scaffolds to the H-λN2 protein displayed on the surface of the transfected HEK 293T cells. The scaffold binding was determined using the Bio oligo in conjunction with the PE-labeled streptavidin (SA-PE). The RNA structure depicted was created from random sequences using the NUPACK Web Application [37]. (**B**) Representative flow cytometry profiles of transfected HEK 293T cells expressing H-λN2, H-IL-13, or H-EGF, and treated with various RNA scaffolds bound to the Bio oligo. The numbers refer to the percentage of PE-positive cells observed with the various scaffolds. Untreated cells: cells not exposed to an RNA scaffold, the Bio oligo or SA-PE.

To display λN protein domains on the HEK 293T cells, a recombinant plasmid encoding two copies of an affinity-enhanced λN protein domain, referred to as λN22 (G1,N2,K4) [35], was used. This domain is referred to here as λN2. The λN2 protein was fused in-frame to the C-terminal end of a mutant version of the MV H protein [20,36]. The resulting fusion protein is referred to as H-λN2. To assess the capacity of the H-λN2 protein displayed on the cells that were to bind to the scaffold RNAs via the boxB sequences, the HEK 293T cells were transiently transfected with an H-λN2-encoding plasmid. The controls included plasmids encoding the MV H protein fused to IL-13 [20] or to epidermal growth factor (EGF). The cells were then exposed to various scaffold RNAs base-paired to a DNA oligonucleotide with complementarity to the scaffold RNA sequences and bearing a biotin group at its 5′ end for the subsequent capturing of Phycoerythrin (PE)-tagged streptavidin (SA) (Figure 1A). This DNA oligonucleotide is referred to as Bio oligo. The flow cytometry results presented in Figure 1B show that the strongest PE signal was obtained using the RNA scaffold Ab2bA. 

Compared to the HEK 293T cells expressing the H-λN2 protein, only a minor increase in the PE signal was observed in the cells transfected with control plasmids expressing the MV H protein fused either to IL-13 or EGF (Figure 1B). Furthermore, the AriBo tag that lacked sequence complementarity with the Bio oligo did not produce a signal above background levels (Figure 1B). These results support the view that scaffold binding to cells was specifically mediated by the H-λN2 protein.

### 2.3. Testing the Capacity of RNA Scaffolds to Bind to the H-λN2 Protein Displayed on Lentiviral Vector Particles

To determine the scaffold binding to the LV vector particles, a mutated version of the MV H protein [20,36] bound to the λN2 protein was used. LV vector particles bearing the H-λN2 protein and containing the RNA scaffolds shown in Figure 1B were immobilized on streptavidin-coated magnetic beads via the Bio oligo.

**Figure 2:**
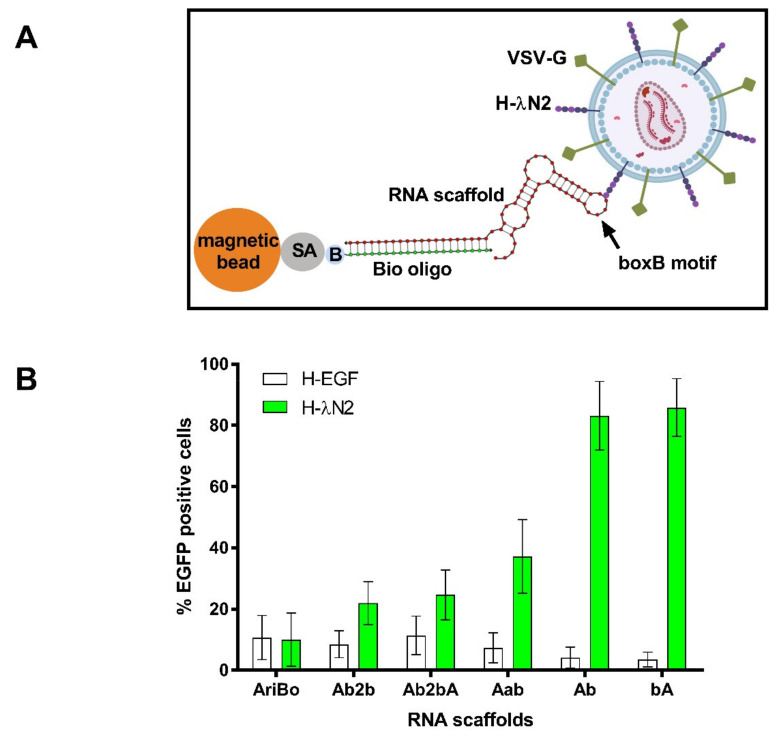
Testing of RNA scaffold binding to the H-λN2 protein displayed on lentiviral vector particles. (**A**) Outline of the approach to test the scaffold binding to the LV vector particles displaying the H-λN2 plus VSV-G proteins. The vectors containing H-EGF instead of H-λN2 were used as a control. The vectors were exposed to RNA scaffolds immobilized on streptavidin-coated magnetic beads via the Bio oligo. (**B**) To determine the transduction efficiencies, magnetic beads with LV vector particles attached were added to A431 cells and the percentage of EGFP-positive cells was determined by using flow cytometry three days later. The error bars represent the means ± SD of three independent experiments.

The EGFP-encoding LV vector particles pseudotyped with the H-λN2 (or H-EGF) and VSV-G glycoproteins were used to monitor the transduction of human skin epidermoid carcinoma epithelial A431 cells (Figure 2A). The percentage of EGFP-positive cells was determined by using flow cytometry. The results presented in Figure 2B show that the highest percentage of EGFP-positive cells was obtained using the bA and Ab scaffolds that bore boxB motifs either at the 5′ (bA) or 3′ (Ab) ends. The percentage of EGFP-positive cells in both cases reached up to 90%. Using the H-EGF-bearing control vector, the percentage of EGFP-positive A431 cells was ~5% (Figure 2B). These results are consistent with the view that the H-λN2 protein displayed on vector particles specifically mediated the binding of the RNA scaffold/Bio oligo complexes to the vector.

### 2.4. RNA Scaffold Binding to RNA Aptamers 

**Figure 3:**
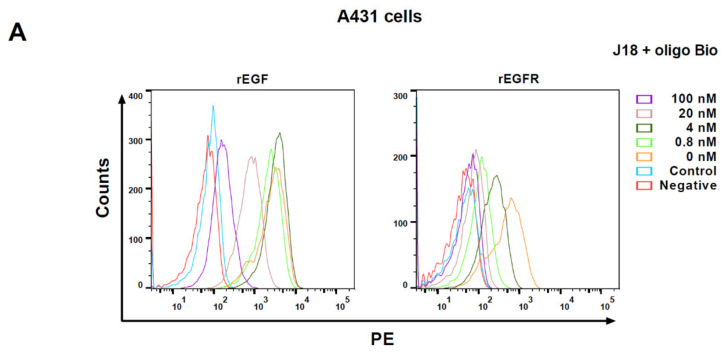

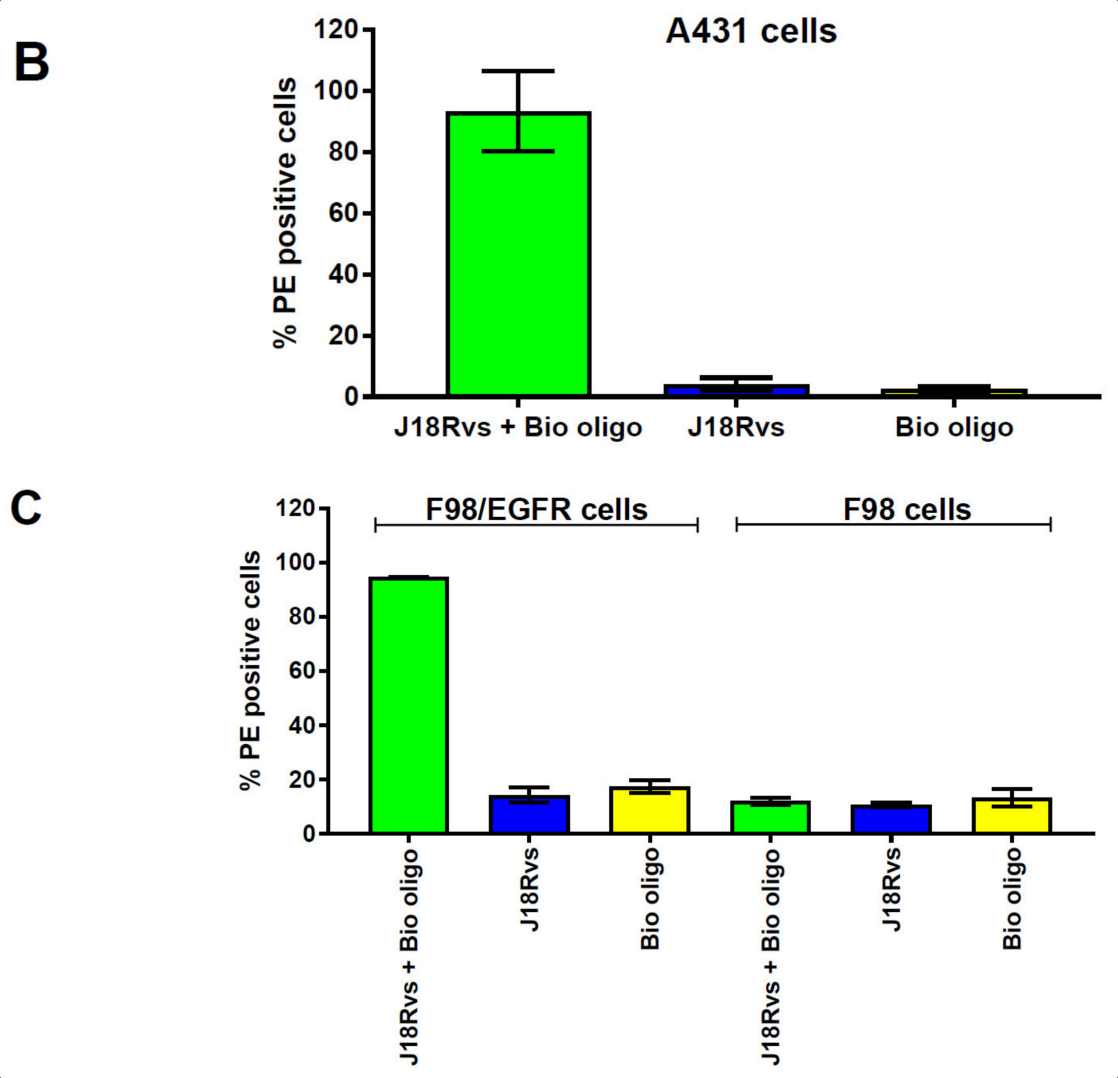
RNA aptamer binding to A431 cells and F98/EGFR cells. (**A**) J18 aptamer binding to A431 cells in the presence of rEGF ranging from 0 nM to 100 nM (left panel) or rEGFR ranging from 0 nM to 100 nM (right panel). Control: SA19 aptamer directed against streptavidin. The aptamer binding was assessed using the oligo Bio bound to the PE streptavidin. Negative: cells not exposed to the J18 aptamer. (**B**) J18 Rvs aptamer binding to A431 cells. The aptamer binding was determined using the Bio oligo bound to the PE streptavidin. (**C**) J18 Rvs aptamer binding to F98/EGFR and F98 cells. Green panels: cells plus J18 Rvs aptamer plus Bio oligo; blue panels: cells plus J18 Rvs aptamer; yellow panels: cells plus Bio oligo. The error bars represent the means ± SD of three independent experiments.

To test the ability of the RNA scaffolds described above to bind to other RNA sequences, a J18 RNA aptamer-based sequence was used. The J18 aptamer was previously shown to bind to human EGFR [38,39,40]. The J18 RNA aptamer was generated by in vitro transcription using the DNA template shown in Figure S2. To test the aptamer’s ability to bind to EGFR, the A431 cell line that expressed high levels of EGFR [41] was used. The presence of EGFR on the A431 cells was confirmed by anti-EGFR antibody binding, and the specificity of the antibody binding was assessed by adding increasing amounts of recombinant EGF (rEGF), recombinant EGFR (rEGFR), or an isotype antibody control (Figure S3). The J18 aptamer was extended at its 3′ end with a sequence (5′ GAAUUAAAUGCCCGCCAUGACCAG 3′) [38], allowing it to bind to a complementary DNA oligonucleotide, referred to as oligo Bio, bearing a biotin group at its 5′ end for the subsequent capture of PE streptavidin. The binding of J18 aptamer/oligo Bio/PE streptavidin complexes to the A431 cells was assessed using flow cytometry. In a manner that was consistent with earlier findings reported by Li et al. [38,39], the flow cytometry analysis showed that aptamer J18 was capable of binding to A431 cells (Figure 3A). Our results also showed that the exposure of the A431 cells to the J18 aptamer in the presence of rEGF or rEGFR resulted in diminished flow cytometry signals (Figure 3A, left and right panels, respectively). This finding is consistent with the view that the J18 aptamer binds to EGFR and that adding soluble rEGFR or rEGF competes with the aptamer binding to cells.

An RNA aptamer (SA19) directed against streptavidin [42] was used as a negative control. It was generated by in vitro transcription, using the DNA template shown in Figure S2. The SA19 aptamer binding produced a flow cytometry signal at background levels (Figure 3A, Control) as did the binding of a scrambled RNA aptamer (results not shown). Furthermore, the co-incubation of the cells with the RiboShredder™ RNase Blend resulted in a complete loss of fluorescence (results not shown), indicating that the flow cytometry signals observed were mediated by the RNA.

The J18 Rvs aptamer, a modified version of the J18 aptamer, was used to assess the scaffold binding. It was generated by in vitro transcription, using the DNA template shown in Figure S2. The results presented in Figure 3B show that the J18 Rvs aptamer retained its capacity to bind to A431 cells, while the scrambled RNA aptamer did not (Figure S4). 

The J18 Rvs aptamer also bound to rat F98 cells expressing human EGFR (referred to as F98/EGFR cells) [43], but not to F98 cells lacking an EGFR-coding region (Figure 3C). The omission of the Bio oligo or the aptamer resulted in PE signals at background levels (Figure 3 B,C).

### 2.5. Assessing the Functionality of RNA Aptamer/Scaffold Complexes

**Figure 4:**
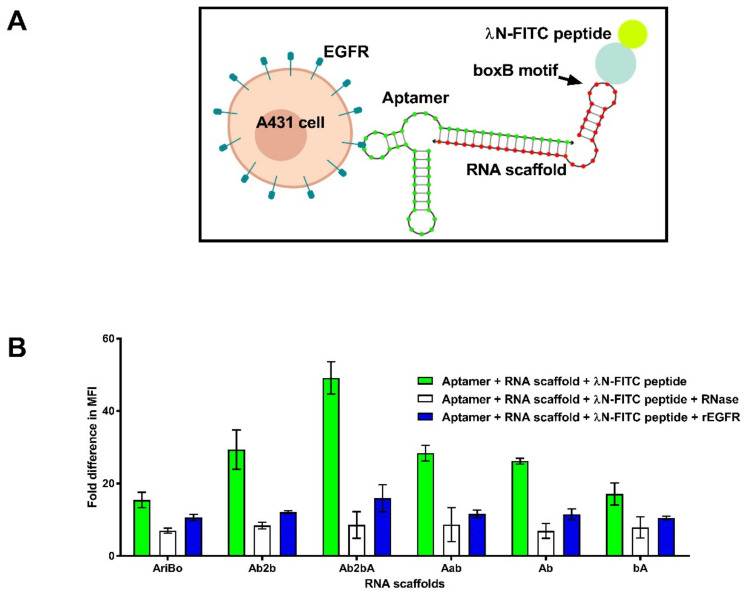
Testing of RNA scaffolds designed to bind the J18 Rvs aptamer and the bacteriophage λN protein. (**A**) Outline of the scaffold binding approach involving the J18 Rvs aptamer and the λN-FITC peptide. (**B**) Binding of aptamer/scaffold/λN-FITC peptide complexes to the A431 cells analyzed by flow cytometry. The green bars refer to the binding to the A431 cells of the J18 Rvs aptamer in combination with an RNA scaffold and the λN-FITC peptide. The open bars refer to samples treated with ~0.02 U/µL of RiboShredder RNase blend prior to flow cytometry. The blue bars refer to the samples treated using rEGFR (1 µM). The binding of the aptamer/scaffold/λN-FITC complexes to the cells was carried out as described in Section 4. The fold differences in MFI refer to the MFI signal obtained with a particular aptamer/scaffold/λN-FITC peptide complex compared to the MFI signal observed with the cells treated with λN-FITC peptide alone. The error bars represent mean ± SD of two independent experiments.

The capacity of the various scaffold RNAs to bind to λN protein sequences was tested next. To do this, aptamer/scaffold complexes were mixed with a synthetic λN peptide bound to FITC (referred to as λN-FITC peptide) (Figure 4A). The A431 cells containing aptamer/scaffold/λN-FITC peptide complexes were subjected to a flow cytometry analysis. For the Ab2bA RNA scaffold, a difference of up to four-fold in the mean fluorescence intensity (MFI) values over those involving cells treated with the λN-FITC peptide alone was observed (Figure 4B), while the differences observed with the Ab2b, Aab, Ab and bA scaffolds were less pronounced (Figure 4B). In the samples treated with rEGFR or RNase or by using the AriBo tag scaffold that lacked a complementary sequence for aptamer binding, the differences in the MFI values observed were smaller (Figure 4B). This result is consistent with the view that the flow cytometry signals observed were due to the binding of the J18 Rvs aptamer/scaffold/λN-FITC peptide complexes to the EGFR on the A431 cells.

The binding specificity of the aptamer/scaffold/λN-FITC peptide complex was assessed in more detail by comparing the ability of the J18 Rvs aptamer/Ab2bA RNA scaffold/λN-FITC complex and the SA19 aptamer/Ab2bA RNA scaffold/λN-FITC complex to bind to A431 cells and to the MDA-MB-435 cells that lacked EGFR [39]. The results presented in Figure S5 show that for the A431 cells treated with the J18 Rvs aptamer/Ab2bA RNA scaffold/λN-FITC complex or the λN-FITC peptide alone, the difference in the MFIs was about four-fold, while for the A431 cells exposed to the SA19 aptamer/Ab2bA RNA scaffold/λN-FITC complex or the λN-FITC peptide alone, there was no difference detected in the MFI values. For the MDA-MB-435 cells treated with the J18 Rvs aptamer/Ab2bA RNA scaffold/λN-FITC complex or the SA19 aptamer/Ab2bA RNA scaffold/λN-FITC, complex there was no difference in the MFIs, indicating that J18 Rvs aptamer binding to the A431 cells was specific.

It was interesting to note that the RNA scaffold binding to the H-λN2 protein displayed on the LV vector particles was highest with the Ab and bA scaffolds (Figure 1B), while the results presented in Figure 4B show that in the context of the transfected A431 cells expressing the H-λN2 protein, the strongest signal was obtained using the RNA scaffold Ab2bA. The reasons for this discrepancy are not clear. There may have been differences in the accessibility of the bulkier Ab2bA RNA scaffold to the H-λN2 protein displayed on the vector particles compared to the transfected A431 cells.

## 3. Discussion

Different protein ligands, such as EGF [19], IL-13 [20,44], single-chain antibodies [21], and designed ankyrin repeat proteins (DARPins) [22], have been successfully displayed on LV vector particles in the past, allowing LV vector delivery to specific target cells in vitro and in vivo [18]. Nucleic acid aptamers capable of binding to a variety of targets, including extracellular ligands and cell surface proteins, have been developed [45,46] and used successfully to mediate the uptake into cells of siRNAs, chemotherapeutic agents, cell toxins, and nanoparticles [47,48], but approaches involving nucleic acid-based aptamers in the retargeting of LV vector transduction have not been described so far. The potential advantage of the nucleic acid aptamer approach over traditional protein ligand approaches is that nucleic acid-based aptamers are easier to prepare and structurally more flexible compared to antibody or peptide-based ligands.

Methods aimed at the attachment of short nucleic acid sequences to LV vector particles in a specific and tight fashion have not been reported thus far. The work described in this study describes a novel approach to the tethering of RNA aptamers to LV vector particles based on the bacteriophage λN/boxB system [49]. The bacteriophage λN/boxB system is attractive because of the small size of the N protein domain (12.2 kDa). Of the 107 amino acids that constitute the N protein, only the 22 amino acid residues of the RNA-binding domain are crucial for RNA recognition [31,32]. A synthetic peptide consisting of these amino acids was found to bind the boxB element with high picomolar affinity and specificity, similar to that of its full-length counterpart [35]. 

The bacteriophage MS2 coat protein system is the most widely used RNA tethering system [50]. The popularity of the bacteriophage MS2 tethering system is based on its physical and functional characteristics: (1) the MS2 coat protein is relatively small (14 kDa, 129 amino acids), (2) it binds its 21-nucleotide RNA hairpin target with high affinity and selectivity, potentially limiting off-target binding, and (3) the MS2 hairpin–MS2 coat protein interaction is well characterized [51]. The potential advantages of the λN/boxB system over other MS2 coat protein systems are that the λN protein domain is smaller and that it binds to RNA as a monomer, unlike the MS2 coat protein, which binds as a dimer [31].

Our results show that the λN/boxB system provides a promising approach to the attachment of short RNA sequences to LV vector particles. In addition, it has the potential to bind short DNA sequences, including DNA aptamers, via a boxB-containing complementary RNA. Our results also show that the binding of the various boxB-containing RNA scaffolds and aptamer sequences to λN protein sequences was specific. In addition, the H-λN2 protein displayed on the LV vector particles, along with the VSV-G, allowed the transduction of A431 cells. Replacing the VSV-G with smaller fusion domains, including the VSV-GS domain [52] may ultimately increase the specificity of this approach.

The limitation of the RNA-capturing approach described in this study is that the high prevalence of antibodies against measles virus proteins may hamper the efficacy of LV vectors containing engineered measles virus H glycoproteins, including H-λN2, in a clinical setting, due to vaccination or natural infection [53]. Glycoproteins from other members of the *Paramyxoviridae* family, including those derived from the Tupaia paramyxovirus (TPMV) have also been used to pseudotype LV vectors [54]. The absence of pre-existing human antibodies positions TPMV glycoproteins as attractive candidates for the design of specific and directed LV vector pseudotyping strategies. In addition, LV vectors pseudotyped with VSV-G are known to be sensitive to complement-mediated inactivation by human serum [26]. The use of alternative vesiculovirus G glycoproteins derived from Cocal virus, Maraba virus or Piry virus may help prevent this pre-existing humoral immunity issue [27]. The impact on the innate response evocated by systemic injection of LV particles exposing short RNAs also needs to be considered [55]. CpG sequences alone or in longer DNA and RNA oligonucleotides can behave like pathogen-associated molecular patterns and trigger an innate immune response, leading to cytokine production via Toll-like receptors. Strategies to ameliorate or eliminate damaging CpG effects have included the methylation of cytosines in the aptamer, the truncation of the aptamer to its essential binding site, and backbone modifications to lessen the toxic CpG effect on innate immunity [56].

In future studies we plan to determine the usefulness of RNA and DNA aptamers tethered to LV vector particles via the λN protein-boxB system for targeted transduction in vitro and in vivo [20,54]. We also plan to investigate whether short RNA and DNA oligonucleotides bound to LV vector particles via the λN protein-boxB system can be used to shield LV vectors against neutralizing antibodies and complement attack. 

## 4. Materials and Methods

### 4.1. Plasmid Constructs

A synthetic DNA sequence (GenScript USA, Piscataway, NJ, USA) encoding the λN2 protein (GNAKTRRRERRAEKQAQWKAANGAGAGAGAGAGAGNAKTRRRERRAEKQAQWKAAN) [33,35] was subcloned into plasmid pCG-HcΔ18-AA-IL-13 [20], replacing the IL-13 coding region. The resulting plasmid is referred to as pCG-HcΔ18-AA-λN2. The DNA sequence encoding human EGF (GenScript USA) was subcloned into pCG-HcΔ18-AA-IL-13 [20], resulting in plasmid pCG-HcΔ18-AA-EGF. Recombinant pUC57-based plasmids encoding the J18 and J18 Rvs and scrambled RNA aptamers, the SA19 aptamer [42], the ARiBo tag [33], and the Ab2b, Ab2bA, Aab, Ab and bA RNA scaffolds, were provided by GenScript USA.

The pNL (CMV)EGFP/CMV/WPREΔU3 LV vector plasmid was described previously [20]. It is available through Addgene, Watertown, MA (plasmid #41970).

### 4.2. Cells

The A431 cells (ATCC CRL-1555, Manassas, VA, USA), MDA-MB-435 cells (ATCC HTB-129), HEK 293T cells (ATCC CRL-3216), F98 cells and F98/EGFR cells (ATCC CRL-2397 and CRL-2948, respectively) were grown in Dulbecco’s Modified Eagle’s Medium (DMEM) containing high glucose (4.5 g/L), 2 mM L-glutamine, 10% heat inactivated fetal bovine serum (FBS), 100 U/mL penicillin and 100 µg/mL streptomycin. The cell culture reagents were purchased from Gibco (Thermo Scientific, Waltham, MA, USA). 

### 4.3. Lentiviral Vector Production

The LV vector production was carried out using 15-cm plates (Nunc, Thermo Scientific, Waltham, MA, USA). One day before transfection, 6 × 10^6^ 293T cells were seeded into each 15 cm plate. The vector production was carried out via PEI-mediated transfection [57] of 7.65 µg of pNL(CMV)EGFP/CMV/WPREΔU3 vector plasmid [20], 5.1 µg pCD/NL-BH*ΔΔΔ packaging plasmid [58], 1.95 µg of pCEF-VSV-G plasmid [20] and 0.6 µg of the pCG-HcΔ18-AA-λN2 or pCG-HcΔ18-AA-EGF plasmids [20] per 15 cm dish. The medium was replaced the following day with 15 mL of Ultraculture medium per 15 cm dish (Lonza, Walkersville, MD, USA), supplemented with 2 mM L-glutamine, 100 U/mL penicillin and 100 µg/mL streptomycin. The LV vector-containing medium was collected 3 days post-transfection and passed through a 0.45 um filter unit. To concentrate the vectors, the medium was mixed 3:1 with Lenti-X™ lentiviral concentrator (Takara Bio USA, Inc., Mountain View, CA, USA). The mixture was incubated at 4 °C for 4 h and then centrifuged at 3000× *g* for 45 min at 4 °C. The pellets were resuspended in 1/10th of the original volume using a 100 mM HEPES buffer (pH 7.5) plus 0.2 U/µL of RNase inhibitor (RiboGuard^TM^, Lucigen, Middleton, WI, USA). By contrast, the vectors were concentrated by ultracentrifugation, as described by Kutner, et al. [59]. 

### 4.4. Production of Aptamer and Scaffold RNAs

The recombinant plasmids encoding the RNA scaffold and the aptamer sequences were linearized using the restriction enzymes referred to in Supplementary Figures S1 and S2 respectively. The in vitro RNA synthesis was carried out for 5 h at 37 °C using an Ampliscribe Kit (Epicentre, Madison, WI, USA). The residual plasmid DNA was degraded using 0.05 U/µL of DNase I (Epicentre) at 37 °C for 15 min. The RNAs were concentrated by ethanol precipitation and fractionated on 8% polyacrylamide gels containing 8 M urea (Thermo Scientific). The RNA bands were visualized by UV shadowing, using Fluor-coated TLC plates (Ambion Inc., Austin, TX, USA). The excised gel bands were frozen at −80 °C for 15 min, crushed using a pellet pestle (Thermo Scientific), and suspended in a TE buffer. Subsequently, the samples were heated at 73 °C for 15 min and a RiboGuard^TM^ RNase inhibitor (Lucigen) (40 U/tube of in TE buffer) was added. The samples were incubated overnight at 37 °C with constant shaking. The extracted RNAs were recovered by filtration, using a 0.45 µm filter (Millipore, Billerica, MA, USA), followed by ethanol precipitation. The RNA pellets were resuspended in the TE buffer. By contrast, the RNAs were purified and concentrated using an RNA Clean & Concentrator^TM^ −5 kit (Zymo Research, Irvine, CA, USA). 

### 4.5. Binding of J18 and J18 Rvs Aptamers to Cells

For cell binding, the J18 RNA aptamer (5′ GGCGCUCCGACCUUAGUCUCUGCAAGAUAAACCGUGCUAUUGACCACCCUCAACACACUUAUUUAAUGUAUUGAACGGACCUACGAACCGUGUAGCACAGCAGAGAAUUAAAUGCCCGCCAUGACCAGAU 3′) was bound to a complementary single-stranded DNA oligonucleotide bearing a biotin group at the 5′ end (IDT, Coralville, IA, USA), using RNA/DNA hybridization. The oligonucleotide used is referred to as oligo Bio (5′ Bio-TGGTCATGGCGGGCATTTAATT 3′). The J18 Rvs aptamer, a modified version of the J18 aptamer, was used to assess the scaffold binding. It consisted of the original J18 aptamer sequence but contained a different sequence (5′ GUGGUCAUGGCGGGCAUUUAAUU 3′) at its 3′ end. To investigate the binding of the J18 Rvs RNA aptamer to the cells, a complementary single-stranded DNA oligonucleotide, referred to as Bio oligo (5′ Bio-AATTAAATGCCCGCCATGACCA 3′) was used. One-to-one molar ratios of the oligo Bio plus the J18 aptamer, or the Bio oligo plus the J18 Rvs aptamer were used. The controls included an RNA aptamer (SA19) directed against streptavidin [42] (5′ GGGAGACAAGACUAGACGCUCAACUUUCCTAGCGCACAUGCGACCUCUAUGCGUAAUACGAACGUUGACGGUUCGACAUGAGACUCACAACAGUUCCCUUUAGUGAGGGUUAAUUCUGGUCAUGGCGGGCAUUUAAUUGA 3′), as well as a scrambled RNA aptamer (5′ GGCGCUCCGACCUUAGUCUCUGUACAGAUCCCAUUCUAUACCCAAAUAACUGUAAAUUAUGACGUACGCCUCCCAUCGAAGAGUGAACCGUGUAGCACAGCAGAGAAUUAAAUGCCCGCCAUGACCAGA 3). 

The formation of RNA/DNA hybrids involved heating the samples for 3 min at 73 °C, with a subsequent gradual decrease in temperature to 25 °C. Next, the RNA/DNA hybrids were incubated at 25 °C for 15 min with a streptavidin R-phycoerythrin conjugate (SA-PE) (Molecular Probes, Life Sciences, Grand Island, NY, USA), using a molar ratio of 2:1. To analyze aptamer binding, A431, MDA-MB-435 cells, F98/EGFR cells or F98 cells were trypsinized using 0.25% Trypsin-EDTA (Thermo Scientific) and washed three times with a DPBS/5 mM MgCl_2_ buffer. For the labeling of the cells, the RNA/DNA/SA-PE complexes were diluted in DPBS/5 mM MgCl_2_ and added to the cells. Binding assays were carried out for 30 min at room temperature in the dark. The samples were then washed with DPBS/5 mM MgCl_2_ and analyzed using flow cytometry.

To assess the specificity of the J18 aptamer binding, rEGFR (ACRO Biosystems, Newark, DE) or rEGF (GenScript USA, Piscataway, NJ, USA) in H_2_O/0.1% BSA were added to compete for aptamer binding to the EGFR. An aptamer that does not bind to EGFR (SA19) was used as an additional control for specificity.

### 4.6. RNA Scaffold Binding to Cells Displaying the H-λN2 Protein

To test the ability of the various RNA scaffolds to bind to MV H-λN2 proteins displayed on the surface of cells, the HEK 293T cells were transfected using the pCG-HcΔ18-AA-λN2 plasmid. The plasmids pCG-HcΔ18-AA-IL-13 and pCG-HcΔ18-AA-EGF were used as controls. The day before transfection, 2 × 10^5^ HEK 293T cells per well were seeded in six well-plates. The next day, the medium was replaced and 1 µg of the pCG-HcΔ18-AA-λN2, pCG-HcΔ18-AA-IL-13 or pCG-HcΔ18-AA-EGF plasmid DNAs in 150 mM were mixed at a 1:1 (*v*/*v*) ratio with 12.6% PEI in 150 mM NaCl, and the DNA/PEI mixture was added to the wells [57]. Forty-eight hours later, the transfected cells were detached from the wells using Ca^++^, and Mg^++^ free PBS containing 0.5 mM EDTA and 50 mM NaCl. The cells were washed three times using PBS and 2 × 10^5^ cells were incubated in the dark for 30 min at room temperature, using a 50 nM solution containing the RNA scaffold base-paired to the Bio oligo, which was bound to SA-PE in a 50 mM HEPES buffer pH 7.5 (total volume: 20 µL). After washing using the 50 mM HEPES buffer, the cells were analyzed using flow cytometry.

### 4.7. Binding of RNA Scaffolds to the J18 Rvs Aptamer and the λN Peptide

The RNA aptamers and scaffolds were mixed and base pairing was initiated via the conditions used for the binding the J18 Rvs aptamer to the Bio oligo. To do this, 100 µL of a 0.15 µM solution containing the J18 Rvs aptamer, plus the RNA scaffold in DPBS/5 mM MgCl_2_, was mixed with 100 µL of a 30 µM solution of the λN peptide conjugated to FITC (FITC-GNAKTRRRERRAEKQAQWKAAN) (Genscript) in DPBS/5 mM MgCl_2_, and incubated for 20 min at 25 °C. For the cell binding, 60 µL of the J18 Rvs aptamer/RNA scaffold/λN-FITC complex were added to 2 × 10^5^ trypsinized A431 or MDA-MB-435 cells and the cells were incubated for 30 min at room temperature in the dark. To determine the specificity of the aptamer binding, 6 μL of a 0.1 µM rEGFR (ACRO Biosystems, Newark, DE) solution in H_2_O/0.1% BSA were added to compete for aptamer binding to the EGFR on the A431 cells. Treatment with 0.02 U/µL of RiboShredder^TM^ RNase Blend (Epicentre) on ice was used to confirm that λN peptide-FITC binding was mediated by the RNA. After incubation for 30 min at room temperature on ice, the cells were washed three times and analyzed using flow cytometry. As an additional control, the SA19 aptamer that does not bind to EGFR was used.

### 4.8. Scaffold Binding to Lentiviral Vectors Displaying the H-λN2 Protein

The lentiviral vector particles bearing H-λN2 proteins were captured using streptavidin-coated magnetic beads (Dynabeads^®^ M-280, Thermo Scientific). To do this, the beads (20 µL/sample) were washed three times with DPBS/5 mM MgCl_2_ and incubated with a ~100 nM solution of a particular RNA scaffold base-paired to the Bio oligo in the presence of 1.3 U/µL of RiboGuard^TM^ (Lucigen). After shaking for 20 min at 1300 rpm at room temperature, a 100 µL aliquot of the magnetic beads containing RNA scaffolds was mixed with a 100 µL aliquot of a LV vector sample displaying either H-λN2 or H-EGF. The titers of the LV vectors displaying the H-λN2 or H-EGF proteins plus VSV-G were typically around 2 x 10^6^ transducing units per ml. The samples were incubated for one hour at 4 °C, with gentle vortexing every 10 min. The samples were then washed three times and the magnetic beads with bound vector particles were diluted in DMEM containing 8 µg/mL polybrene and added to the A431 cells in six wells-plates, seeded the day before, at a density of ~1 × 10^5^ cells/well. The percentage of transduced cells based on EGFP expression was determined using flow cytometry three days later [59].

## Data Availability

The data presented in this study are available in the article and Supplementary Materials.

## Supplementary Materials

The following are available online at https://www.mdpi.com/article/10.3390/ijms221910263/s1.

## Abbreviations

EGFR	Epidermal growth factor receptor
EGF	Epidermal growth factor
IL-13	Interleukin-13
LV	Lentiviral
MV	Measles virus
SA	Streptavidin
PE	Phycoerythrin
VSV-G	Vesicular stomatitis virus G protein
