# Supplementary material for: Tagging and Capturing of Lentiviral Vectors Using Short RNAs
Source: Int J Mol Sci. 2021 Sep 23;22(19):10263. doi: 10.3390/ijms221910263 (PMC8508951; doi:10.3390/ijms221910263)
Supplement: Supplementary file 1 [file ijms-22-10263-s001.zip › Figure S1.pdf]

AriBo RNA scaffold

1 TAATACGACT CACTATAGCG AGTATAACCT CAATAATATG GTTTGAGGGT
51 GTCTACCAGG AACCGTAAAA TCCTGACTAC TCGCGAGCGC CGAACTGG**GC**
101 **CCTGAAGAAG GGC**TCAGTTG ACGAGGTGGG GTTTATCGAG ATTTCGGCGG
151 ATGACTCCCG GTTGTTCATC ACAACCGCAA ACTTTTACTT AAATCATTAA
201 GGTGACTTAG TGGACAAAGG TGAAAGTGTG ATGAATTCAC TGATTGGATC
251 GGATCC

Ab2b RNA scaffold

1 TAATACGACT CACTATAGGG AATTAAATGC CCGCCATGAC CAAGCGCCGA
51 ACTGG**GCCCT GAAGAAGGGC** TCAGTTGACG AGGTGGGGTT TATCGAGATT
101 TCGGCGGATG ACTCCCGGTT GTTCATCACA ACCGCAAACT TTTACTTAAA
151 TCATTAAGGT GACTTAGTGG ACAAAGGTGA AAGTGTGATG AATTCCCTTG
201 G**GCCCTGAAG AAGGGC**TCTT CTCTC**GCCCT GAAGAAGGGC** GGAAGCTTAT
251 TGGATCGGAT CC

Ab2bA RNA scaffold

1 TAATACGACT CACTATAGGG AATTAAATGC CCGCCATGAC CAAGCGCCGA
51 ACTGG**GCCCT GAAGAAGGGC** TCAGTTGACG AGGTGGGGTT TATCGAGATT
101 TCGGCGGATG ACTCCCGGTT GTTCATCACA ACCGCAAACT TTTACTTAAA
151 TCATTAAGGT GACTTAGTGG ACAAAGGTGA AAGTGTGATG AATTCCCTTG
201 G**GCCCTGAAG AAGGGC**TCTT CTCTC**GCCCT GAAGAAGGGC** GGAATTAAAT
251 GCCCGCCATG ACCAAGCTTA TCTAGA

Aab RNA scaffold

1 TAATACGACT CACTATAGGG AATTAAATGC CCGCCATGAC CAAGCGCCGA
51 ACTGG**GCCCT GAAGAAGGGC** TCAGTTGACG AGGTGGGGTT TATCGAGATT
101 TCGGCGGATG ACTCCCGGTT GTTCATCACA ACCGCAAACT TTTACTTAAA
151 TCATTAAGGT GACTTAGTGG ACAAAGGTGA AAGTGTGATG AATTCAAGCT
201 TATTGGATCG GATCC

Ab RNA scaffold

1 TAATACGACT CACTATAGGG AATTAAATGC CCGCCATGAC CAGA**GCCCTG**
51 **AAGAAGGGC**G AAGCTT

bA RNA scaffold

1 TAATACGACT CACTATA**GCC CTGAAGAAGG GC**GAAATTAA ATGCCCGCCA
51 TGACCAGAAG CTT

Figure S1: DNA templates used for scaffold RNA synthesis. The singly underlined sequence refers to the T7 promoter. The doubly underlined sequence provides complementarity for binding a biotin tagged DNA oligonucleotide or the J18 Rvs aptamer. The boxB RNA-encoding sequences are shown in bold. The dotted line refers to the cleavage sites of the restriction enzymes used to linearize the scaffold templates. BamHI was used for the AriBo, Ab2b and Aab templates, XbaI for the Ab2bA template, and HindIII for the Ab and bA templates.
