# Supplementary material for: Tagging and Capturing of Lentiviral Vectors Using Short RNAs
Source: Int J Mol Sci. 2021 Sep 23;22(19):10263. doi: 10.3390/ijms221910263 (PMC8508951; doi:10.3390/ijms221910263)
Supplement: Supplementary file 1 [file ijms-22-10263-s001.zip › Figure S2.pdf]

J18 aptamer

1 TAATACGACT CACTATAGGC GCTCCGACCT TAGTCTCTG***C AAGATAAACC***
51 ***GTGCTATTGA CCACCCTCAA CACACTTATT TAATGTATTG AACGGACCTA***
101 ***C***GAACCGTGT AGCACAGCAG AGAATTAAAT GCCCGCCATG ACCAGAAGCT T

J18 Rvs aptamer

1 TAATACGACT CACTATAGGC GCTCCGACCT TAGTCTCTGC ***AAGATAAACC***
51 ***GTGCTATTGA CCACCCTCAA CACACTTATT TAATGTATTG AACGGACCTA***
101 ***C***GAACCGTGT AGCACAGCAG AGTGGTCATG GCGGGCATTT AATTGACGCG T

Scrambled aptamer

1 TAATACGACT CACTATAGGC GCTCCGACCT TAGTCTCTG***T ACAGATCCCA***
51 ***TTCTATACCC AAATAACTGT AAATTATGAC GTACGCCTCC CATCGAAGAG***
101 ***T***GAACCGTGT AGCACAGCAG AGAATTAAAT GCCCGCCATG ACCAGAAGCT T

SA19 aptamer

1 TAATACGACT CACTATAGGG AGACAAGACT AGACGCTCAA ***CTTTCCTAGC***
51 ***GCACATGCGA CCTCTATGCG TAATACGAAC GTTGACGGT***T CGACATGAGA
101 CTCACAACAG TTCCCTTTAG TGAGGGTTAA TTCTGGTCAT GGCGGGCATT
151 TAATTCAAGC TT

Figure S2: DNA templates used for in vitro RNA synthesis of the J18, J18 Rvs, scrambled and SA19 aptamers. The DNA sequences encoding the J18, J18 Rvs, scrambled and SA19 aptamers are shown in bold. The doubly underlined sequences provide complementarity for binding to a biotin tagged DNA oligonucleotide. The singly underlined sequence refers to the T7 promoter. The dotted line refers to the cleavage sites of the restriction enzymes used to linearize the plasmids. The DNA template used for the J18 Rvs aptamer was linearized using MluI while HindIII was used to linearize the other templates.
