# Supplementary material for: Tagging and Capturing of Lentiviral Vectors Using Short RNAs
Source: Int J Mol Sci. 2021 Sep 23;22(19):10263. doi: 10.3390/ijms221910263 (PMC8508951; doi:10.3390/ijms221910263)
Supplement: Supplementary file 1 [file ijms-22-10263-s001.zip › Figure S4.pdf]

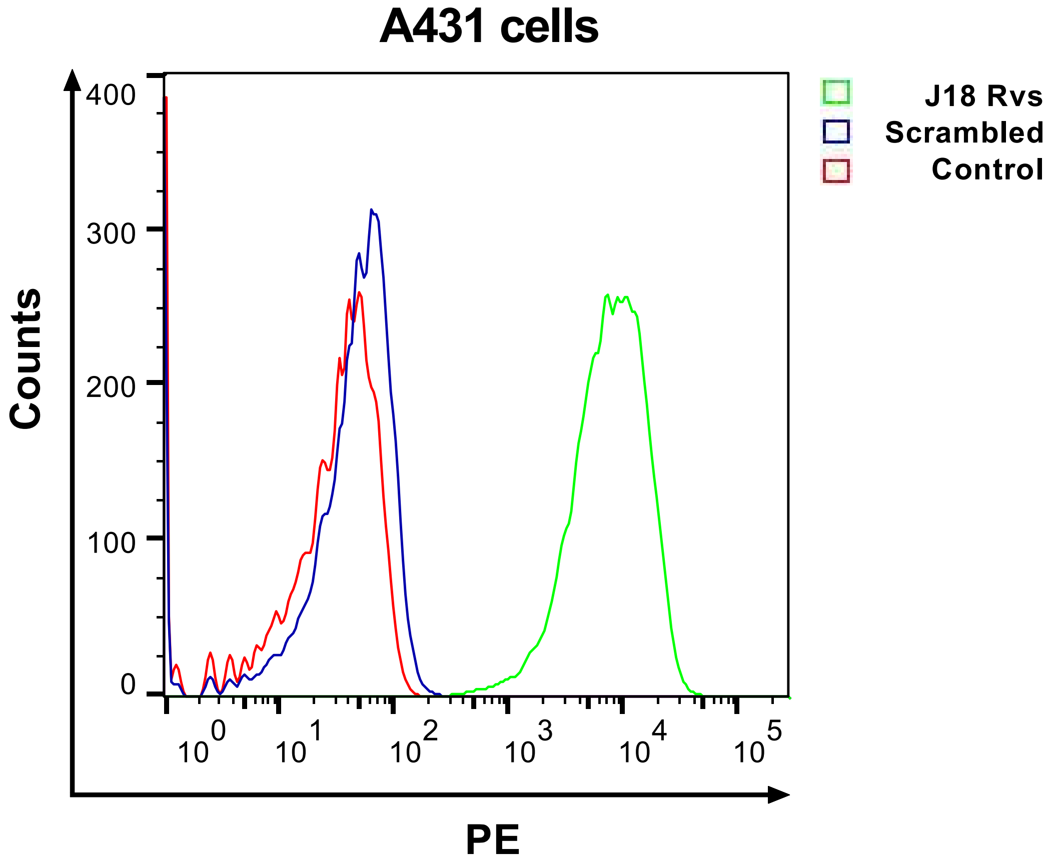

Figure S4: Analysis of binding to A431cells of the J18 Rvs RNA aptamer and a scrambled RNA aptamer. Control refers to A431 cells not exposed to the aptamer. The blue peak represents cells exposed to the J18 Rvs aptamer plus Bio oligo bound to PE streptavidin; The green peak represents cells exposed to the scrambled aptamer plus PE streptavidin. The FACS analysis presented is representative of several performed.
