# Supplementary material for: Tagging and Capturing of Lentiviral Vectors Using Short RNAs
Source: Int J Mol Sci. 2021 Sep 23;22(19):10263. doi: 10.3390/ijms221910263 (PMC8508951; doi:10.3390/ijms221910263)
Supplement: Supplementary file 1 [file ijms-22-10263-s001.zip › Figure S5.pdf]

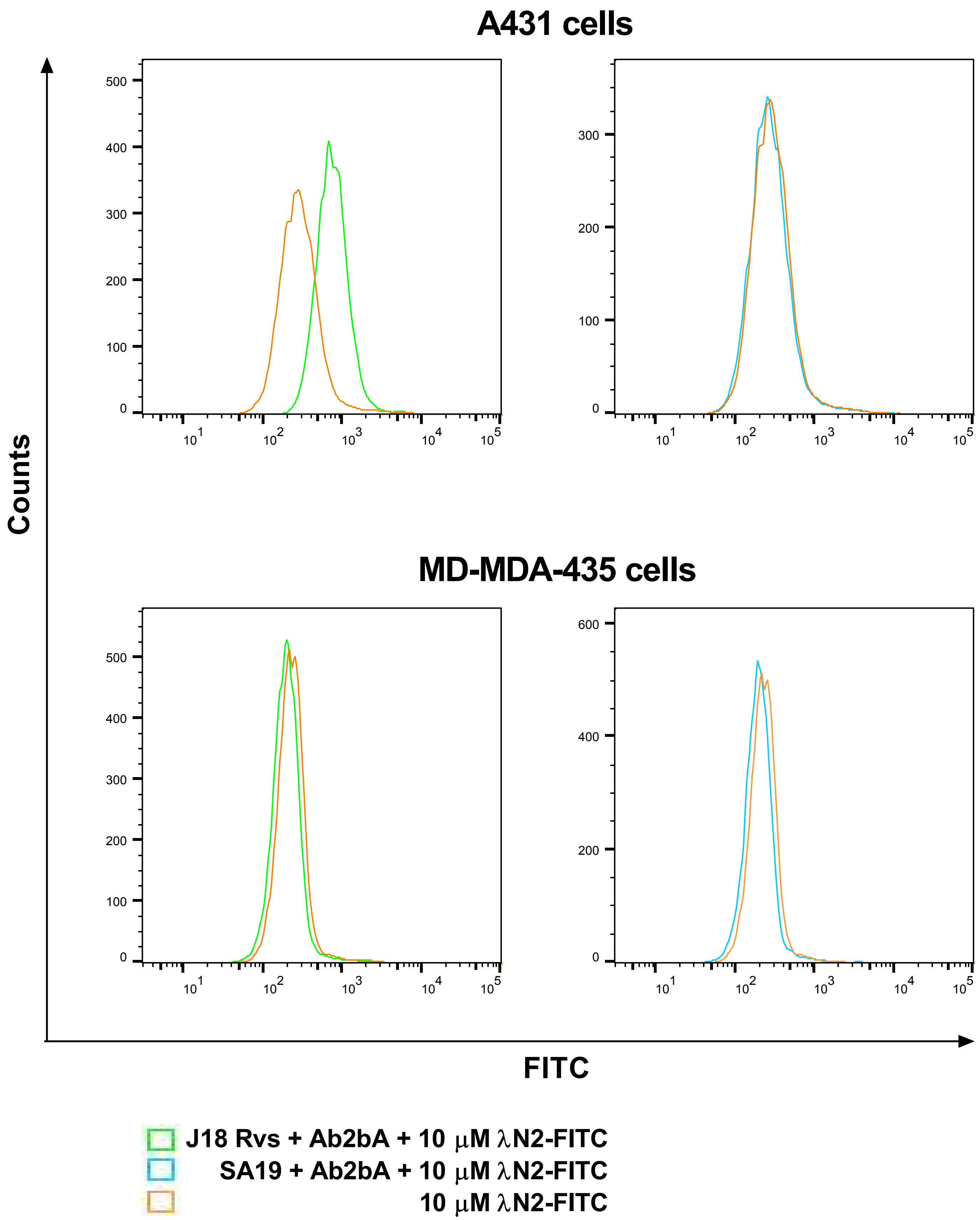

Figure S5: Specificity of binding of J18 Rvs aptamer and of bacteriophage λN-FITC peptide. A431 cells (top panels) and MDA-MB-435 cells (bottom panels) were exposed to J18 Rvs aptamer/Ab2bA RNA scaffold/λN-FITC complexes (green lines) or SA19 aptamer/Ab2bA RNA scaffold/λN-FITC complexes (blue lines). Samples were analyzed by FACS.
