# Supplementary figures and images for: Tagging and Capturing of Lentiviral Vectors Using Short RNAs
Source: Int J Mol Sci. 2021 Sep 23;22(19):10263. doi: 10.3390/ijms221910263 (PMC8508951; doi:10.3390/ijms221910263)

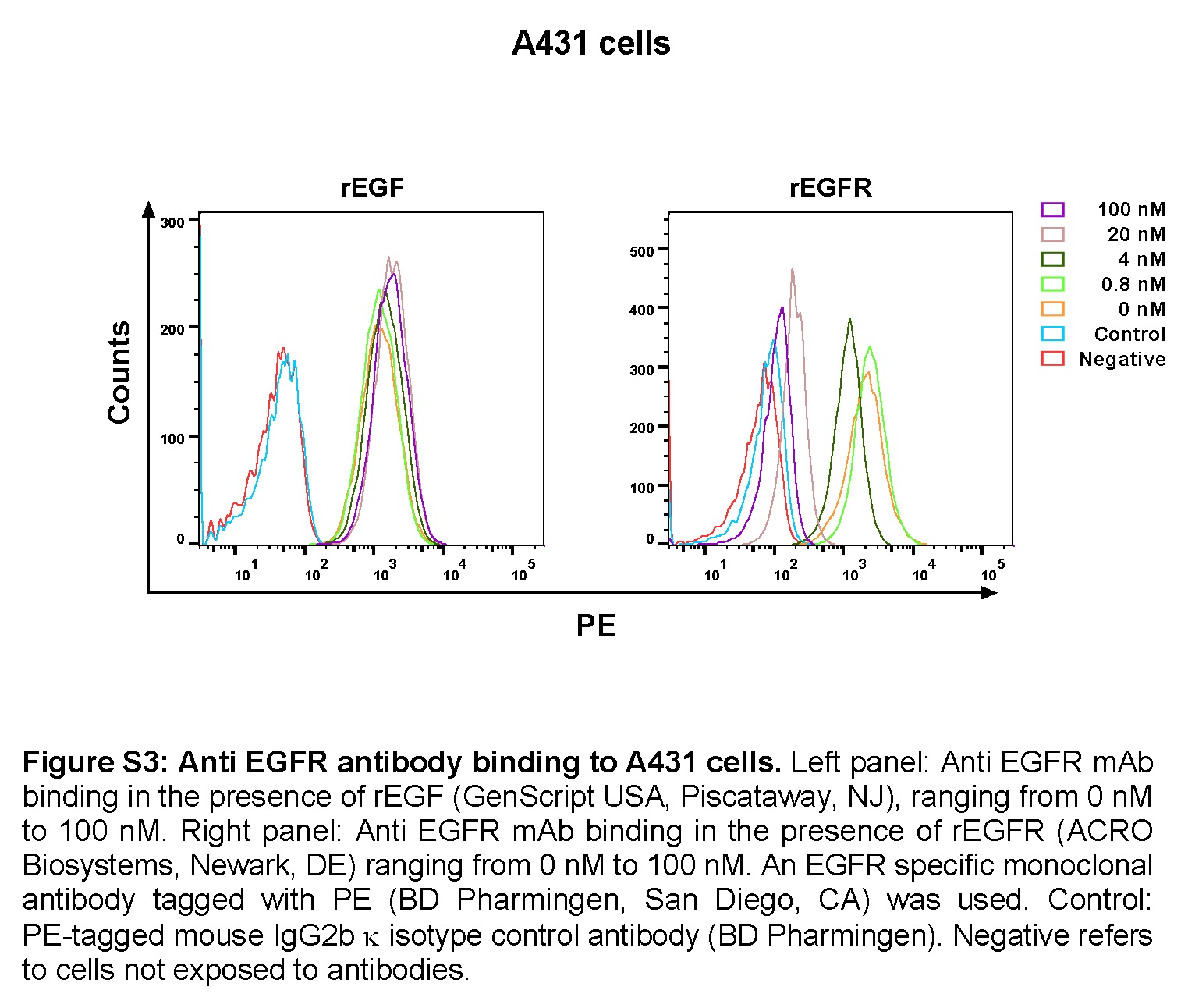

Supplement: Supplementary file 1 [file ijms-22-10263-s001.zip › Figure S3 (revised).jpg]
